# State Space Model with hidden variables for reconstruction of gene regulatory networks

**DOI:** 10.1186/1752-0509-5-S3-S3

**Published:** 2011-12-23

**Authors:** Xi Wu, Peng Li, Nan Wang, Ping Gong, Edward J Perkins, Youping Deng, Chaoyang Zhang

**Affiliations:** 1 School of Computing, University of Southern Mississippi, Hattiesburg, MS 39406, USA; 2 Laboratory of Molecular Immunology, National Heart Lung and Blood Institute, National Institutes of Health, Bethesda, MD 20892, USA; 3 Environmental Services, SpecPro Inc., San Antonio, TX 78216, USA; 4 Environmental Laboratory, U.S. Army Engineer Research and Development Center, Vicksburg, MS 39180, USA; 5 Department of Internal Medicine, Rush University Medical Center, Chicago, IL 60612, USA

## Abstract

**Background:**

State Space Model (SSM) is a relatively new approach to inferring gene regulatory networks. It requires less computational time than Dynamic Bayesian Networks (DBN). There are two types of variables in the linear SSM, observed variables and hidden variables. SSM uses an iterative method, namely Expectation-Maximization, to infer regulatory relationships from microarray datasets. The hidden variables cannot be directly observed from experiments. How to determine the number of hidden variables has a significant impact on the accuracy of network inference. In this study, we used SSM to infer Gene regulatory networks (GRNs) from synthetic time series datasets, investigated Bayesian Information Criterion (BIC) and Principle Component Analysis (PCA) approaches to determining the number of hidden variables in SSM, and evaluated the performance of SSM in comparison with DBN.

**Method:**

True GRNs and synthetic gene expression datasets were generated using GeneNetWeaver. Both DBN and linear SSM were used to infer GRNs from the synthetic datasets. The inferred networks were compared with the true networks.

**Results:**

Our results show that inference precision varied with the number of hidden variables. For some regulatory networks, the inference precision of DBN was higher but SSM performed better in other cases. Although the overall performance of the two approaches is compatible, SSM is much faster and capable of inferring much larger networks than DBN.

**Conclusion:**

This study provides useful information in handling the hidden variables and improving the inference precision.

## Introduction

Microarrays can simultaneously measure the expression of thousands of genes. In the past decade or so, many time series experiments have employed microarrays to profile the temporal change of gene expression. For instance, one can retrieve many time-course gene expression datasets from the Gene Expression Omnibus database (http://www.ncbi.nlm.nih.gov/geo/). These datasets usually have much smaller numbers of time points, compared to the large number of genes. Here we focus on how to infer gene regulatory networks (GRNs) from time series microarray datasets.

Any effective GRN inference method has to cope well with the large number of genes and the small number of time points that characterize microarray datasets. During the past few decades, many methods have been developed, such as Dynamic Bayesian Network (DBN) [1,2] and Probability Boolean Network (PBN) [3]. However, DBN and PBN cannot be used to infer large networks with hundreds of genes due to computational overhead. Thus, there is a need to study different approaches to improving inference accuracy and reducing computational cost.

A State Space Model (SSM) [4-8] has been developed for GRN inference in recent years. It has attracted much attention because it has a much higher computational efficiency and can handle noise well. The variables in SSM can be divided into two groups, hidden variables and observed variables. Observed variables are expression levels of genes measured by microarray experiments. Hidden variables include aspects of the evolution process.

In this study, we investigated the performance of SSM and addressed the effect of the number of hidden variables on inference accuracy. An intuitive way is to let the number of hidden variables equal that of observed variables, but SSM may not be convergent. To make it feasible to infer a large network from a limited number of time points, we need to determine the number of hidden variables in SSM. [4,6,7] used Bayesian Information Criterion (BIC), [5] used cross-validation and [9,10] used Principal Component Analysis (PCA) to determine the number of hidden variables. These methods give a unique value for the number of hidden variables under their corresponding optimal definitions. However, since we are mostly interested in inference of GRNs, one should use accuracy of inferred GRNs to define the optimal criteria. That is, the optimal number of hidden variables that leads to the highest accuracy. It is found that PCA and BIC approaches do not necessarily produce an optimal number of hidden variables. Instead, simply setting the number of hidden variables may give a better or compatible accuracy in SSM. To evaluate the overall performance of SSM with hidden variables, we inferred a number of GRNs using synthetic datasets with different numbers of genes and time points generated from GeneNetWeaver [11].

## Methods

In this section, we briefly present the SSM method and two approaches (BIC and PCA) for determining the number of hidden variables in GRN inference.

### State Space Model

There are two kinds of variables in SSM [12-14], hidden variables *xt* with dimension *m *and observed variables *y_t _*with dimension *l*. SSM consists of system and observation equations:

(1) $$\begin{array}{c} x_{t}=Fx_{t-1}+w_{t}\\ y_{t}=Hx_{t}+v_{t}. \end{array}$$

*w_t _*and *v_t _*are Gaussian noise term. *F *is a state transition matrix. *H *is an observation matrix. Matrices *F *and *H *can be used to determine GRN [7,14]:

(2) $$C=HF{\left(H^{\prime }H\right)}^{-1}H^{\prime }.$$

We used expectation-maximization (EM) [12,15] to infer parameters in SSM.

### Bayesian Information Criterion

As mentioned above, how to determine the number of hidden variables is an important factor affecting the accuracy of inferred GRNs. [4,6,7] used BIC to accomplish this task. We will demonstrate that, BIC cannot give the optimal solution. According to [12], BIC is defined as follows:

(3) BIC= lnP(xt,yt|θ)-12Nθ lnN.

*P*(*x_t_*,*y_t_*|*θ*) is probability given parameter *θ*; *N_θ _*is the number of parameters; *N *is the number of data points. BIC can be calculated with a given number of hidden variables. The number of hidden variables that has the largest BIC will be adopted as the optimal solution.

### Principal Component Analysis

Because the number of time points is usually much smaller than the number of genes, a microarray dataset *y_t_*(*t *= 1,...*T*) has redundant information. From another aspect of view, all measurements for *i*-th gene form a vector of length *T*, *g_i_*. *g_i_*(*i *= 1,...*l*) form a linear space, whose dimension is less than or equal to min(*l*,*T*) [12]. Vectors *g_i _*and *g_j_*(*i *≠ *j*) may not be orthogonal. Here inner product is defined as covariance between those two vectors. One can find a new set of orthogonal bases, *z_k_*(*k *= 1,... min(*l*,*T*)), and *g_i _*can be expressed as linear combination of *z_k_*, since they belong to the same linear space. If one only uses a fraction of new bases, for example, *z_k_*(*k *= 1,...*m*, *m *< min(*l*,*T*), then *g_i _*cannot be fully recovered. However, one can choose the most important *z_k_*, to let the error be minimized. This can be done by using PCA [9,10,12]. Roughly speaking, the error $d=\overset{\text{min}\left(l,T\right)}{\underset{k=m+1}{\sum }}\lambda_{k}$. *λ_k _*is eigenvalue of covariance matrix of dataset *g_i_*. If one throws away those bases *z_k _*whose *λ_k _*are small, then the dimension of microarray dataset is reduced. One must notice that, this method of dimension reduction is approximate due to the small amount of time points. For example, if there are 10 genes with only 1 time point, then one possible way to extract GRN is that, if the expression levels of gene *i *and *j *both are large, then one expect there is a regulatory relationship between them. This means that the dimension of linear space of *g_i_*(*i *= 1,...*l*) is 1, even though the real dimension is not 1. Due to the lack of time points, treating the dimension as 1 is the best way to extract a GRN.

SSM uses the same idea as PCA does [12]. The second equation of (1) contains dimension reduction. The dimension of hidden variables *x_t _*is less than *y_t_*. BIC [4,6,7] and PCA [9,10] can be used to determine the dimension of *x_t_*.

## Results and discussion

Two types of synthetic datasets generated by using GeneNetWeaver [11] were used as test cases in this paper, one for *E. coli *and the other for yeast. We generated 10 GRNs with 30 genes and 41 time points for each of them. The purpose of generating 10 GRNs is to eliminate errors due to particular network topology or attributes, since a GRN inference algorithm may perform better for some GRNs than for the others.

**Figure 1:**
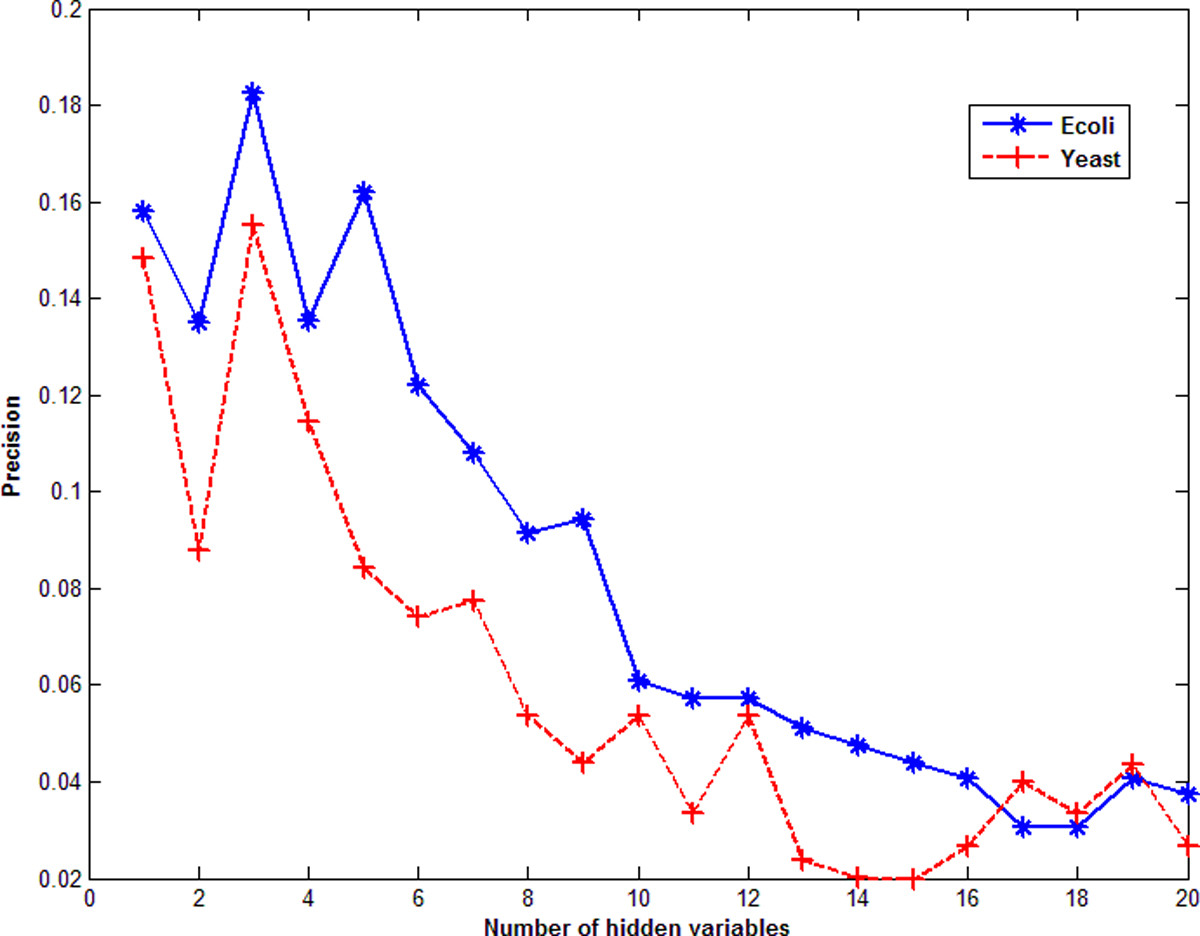
**The relationship between precision and the number of hidden variables by using SSM with *E. coli *and yeast datasets**.

**Figure 2:**
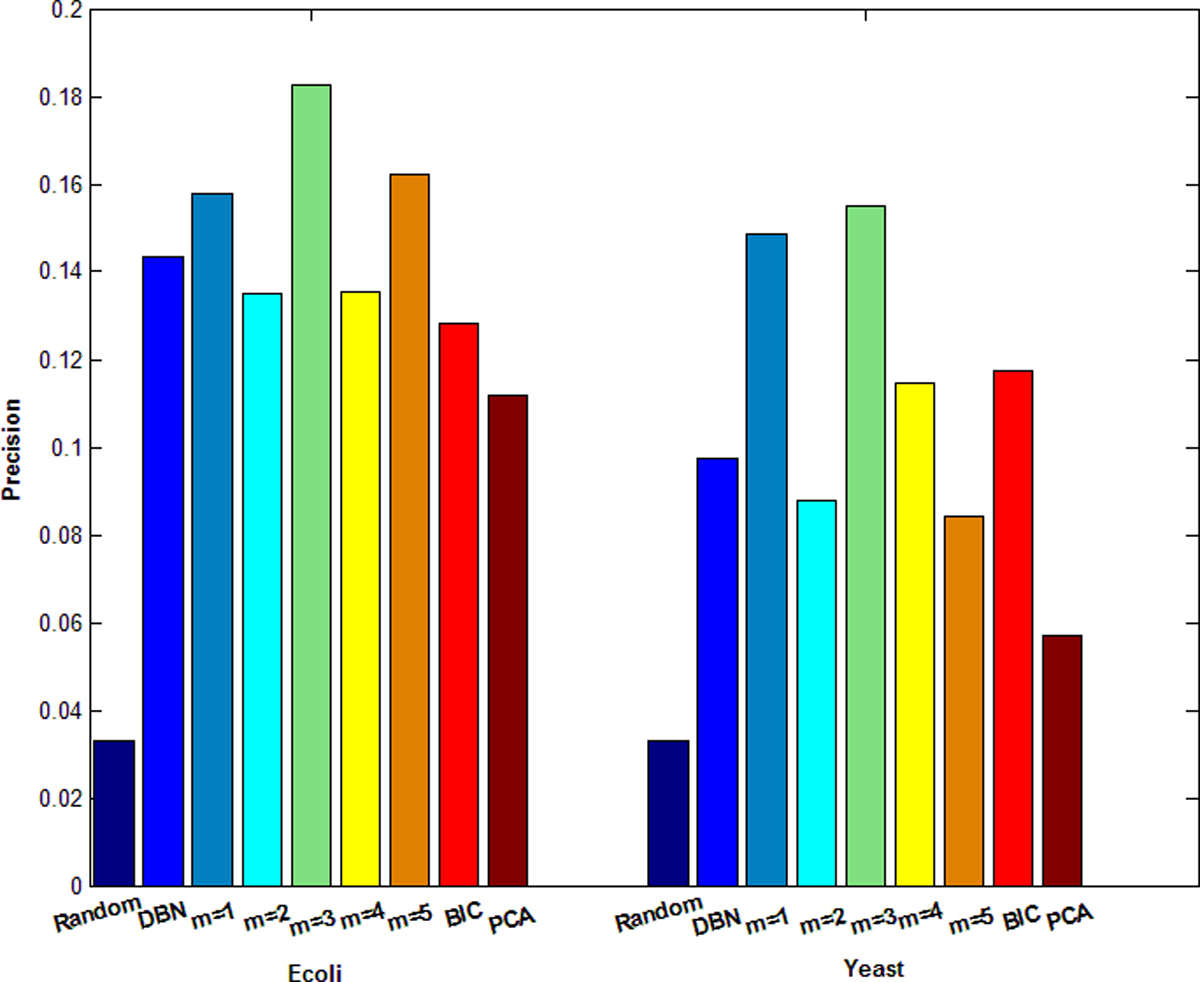
**Precisions of GRNs inferred by SSM and DBN from synthetic Ecoli and Yeast datasets, respectively**. 'Random' means using random guess. 'm = 1' means that the number of hidden variables is set to 1 in SSM. 'm = 2,..., 5' have similar meanings. The first and second halves of figure 2 are for Ecoli and Yeast datasets, respectively.

We only compared the precision of GRNs inferred by SSM with that by the time-delayed DBN. The reason is that the precision of time-delayed DBN is higher than traditional DBN by considering transcriptional time lag [2] and DBN, referred as the time-delayed DBN hereafter, performs a little better than PBN [16]. Here, precision is defined as true positive edges over total number of edges in an inferred GRN. For the convenience of comparison, the number of edges inferred by SSM is set to be the same as that inferred by DBN, so the comparison of precision is equivalent to the comparison of number of true positive edges inferred by SSM and DBN. Because precisions are different for the 10 GRNs of *E. coli *or yeast, we choose to compare the average precision of those 10 GRNs. The number of hidden variables *m *determined by PCA is the first *m *satisfying $\overset{\min\left(l,T\right)}{\underset{k=m+1}{\sum }}\lambda_{k}∕\overset{\min\left(l,T\right)}{\underset{k=1}{\sum }}\lambda_{k}\leq 20\%$. Results are shown in Figures 1 and 2. Figure 1 shows the variation of average precision inferred by SSM over the number of hidden variables for ten *E. coli *or yeast datasets. One can see that, if the number of hidden variables is set between 1 and 5, the corresponding precision is high. If the number of hidden variables is larger than 6, the precision decreases. In Figure 1, the number of hidden variables was simply set the same for all 20 networks. However, it may change for different networks. BIC [4,6,7] and PCA [9,10] can be applied to determine the number of hidden variables. Figure 2 gives the precisions of inferred GRNs (with directed edges) obtained by SSM with a fixed number of hidden variables or determined by BIC and PCA on *E. coli *and yeast datasets, as well as the result by DBN. The results show that neither BIC nor PCA gives a better precision than a fixed number between 1 and 5. Among the 10 *E. coli *datasets, SSM gives higher precision scores (when *m *= 2) for 6 datasets than DBN. Among the 10 yeast datasets, SSM gives higher precision scores (when *m *= 2) for 4 datasets than DBN and the same precision scores as DBN for 2 datasets. The overall performance of SSM when *m *= 3 is much better than that of DBN, as shown in Figure 2. Overall, SSM has better or compatible performance than DBN.

**Figure 3:**
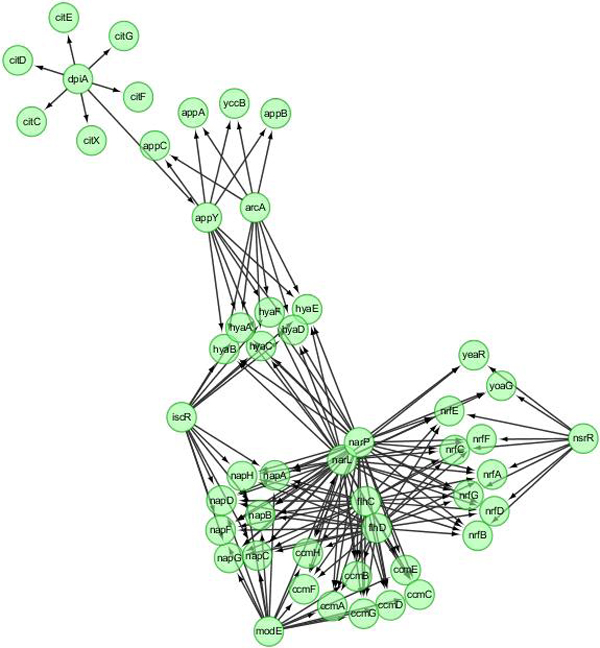
**A true *E. coli *network with 50 genes and 169 edges generated from GeneNetWeaver**.

**Figure 4:**
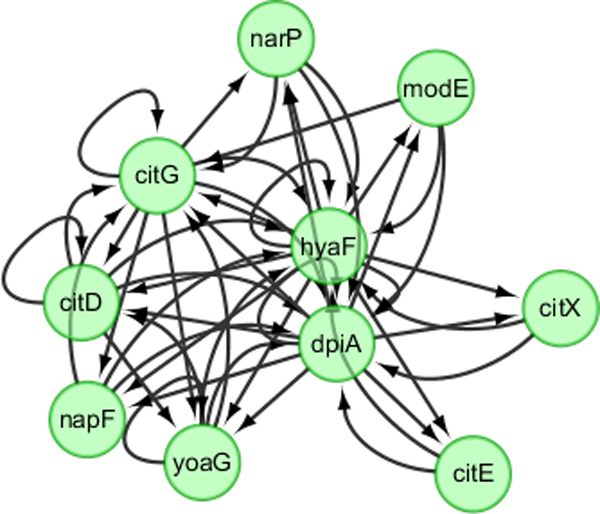
**Inferred GRN with 50 edges by using SSM with 2 hidden variables**.

The precision of GRN inferred by SSM or DBN may depend on network size and the number of time points. To systematically compare the performance of SSM and DBN, we generated synthetic datasets of 10 networks, each with 50 genes and 101 time points, for Ecoli and Yeast, respectively. One true Ecoli network and networks inferred using SSM and DBN are shown in Figure 3, 4, 5. Those networks are drawn using Cytoscape [17]. Both SSM and DBN can correctly infer 5 edges out of total 50 edges. SSM successfully identifies the hub gene *dpiA *and DBN successfully identifies the hub gene *appY*. For Ecoli datasets, average precision scores of 10 networks are 9.2% and 5.9% for SSM with *m *= 2 and DBN. For Yeast datasets, average precision scores are 8.5% and 7.5% for SSM with *m *= 2 and DBN. The result obtained from a larger network with 50 genes also shows that SSM with a fixed number of hidden variables (here *m *= 2) gives a better or compatible precision than DBN. In a typical case, computational times are 4 and 40 seconds for SSM and DBN, respectively, where the number of maximum parents for each gene is set as 3 in DBN. If more parents (regulators) are set for one gene, the computational time of DBN will increase significantly.

**Figure 5:**
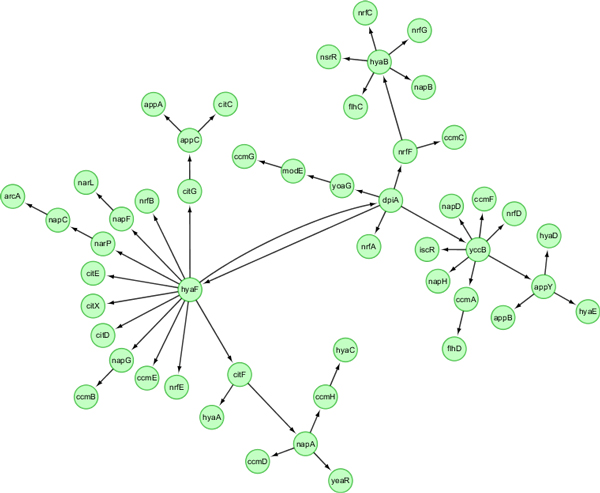
**Inferred GRN with 50 edges by using DBN**.

It is worthwhile to note that when the number of hidden variables is small, some regulations are bidirectional in GRNs obtained by SSM, which means gene *i *regulates gene *j *and in the same time gene *j *regulates gene *i*. This is because the number of hidden variables in SSM is small (= 2 here).

**Figure 6:**
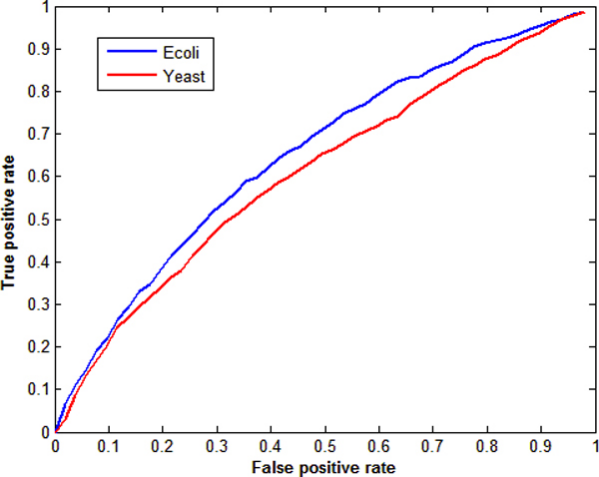
**ROC curve for *E. coli *and yeast datasets with 50 genes by using SSM with 2 hidden variables**. The false and true positive rates are averaged rates over 10 corresponding GRNs.

Another advantage of SSM compared with DBN is that SSM can adjust the number of edges in the inferred GRN. DBN always chooses the network that gives the highest score, whose number of edges is definite. From equation (2) one can see that, the network given by SSM is a matrix *C*. Then one can define a threshold number *th*; *abs*(*C_ij_*) ≥ *th *will lead to an edge (gene *j *regulates gene *i*) [8,14]. Adjusting *th*, one can get networks with different number of edges. If the number of edges is small, precision is higher and recall is lower. If the number of edges is large, precision is lower and recall is higher. Receiver Operating Characteristic (ROC) curve [8,18] is used to demonstrate the performance of inference algorithms. Figure 6 shows results for Ecoli and Yeast datasets with 50 genes, by using SSM with 2 hidden variables. Those ROC curves are above the diagonal, showing that SSM is better than the random guess.

## Conclusions

Determining the number of hidden variables in SSM is important in GRN inference. Our results using synthetic time series gene expression datasets of *E. coli *and yeast, generated by GeneNetWeaver, show that the existing BIC and PCA approaches may not be able to determine the optimal number of hidden variable in SSM. None of them can lead to a better performance than simply setting a fixed number of hidden variables (between1 and 5). In all the tested cases, the average precision scores of GRNs inferred by SSM are mostly better than or compatible with that of DBN. SSM is much more computationally efficient than DBN, enabling the inference and analysis of larger GRNs.

## List of abbreviations used

SSM: State Space Model; DBN: Dynamic Bayesian Networks; GRNs: Gene regulatory networks; BIC: Bayesian Information Criterion; PCA: Principle Component Analysis; PBN: Probability Boolean Network.

## Competing interests

The authors declare that they have no competing interests.

## Authors' contributions

JZ, PG and YD initiated the project. WX and PL developed and implemented the algorithms. WX and JZ performed in-depth analysis of results and drafted the paper. PG, NW and EJP participated in network inference and analysis. PG, EJP and YD revised the paper. All authors read and approved the final manuscript.
